# Mechanistic Investigation of Vitexin in Ameliorating Ovarian Fibrosis in PCOS Mice via the NR4A1/NLRP3 Signaling Pathway

**DOI:** 10.3390/metabo16050332

**Published:** 2026-05-15

**Authors:** Haoran Sun, Jiejing Xu, Chengxue Pan, Jia-Le Song, Yanyuan Zhou

**Affiliations:** 1 Guangxi Key Laboratory of Diabetic Systems Medicine, Guilin Medical University, Guilin 541199, China; shran@stu.glmu.edu.cn (H.S.); xujiejing@stu.glmu.edu.cn (J.X.); 2 Guangxi Key Laboratory of Drug Discovery and Optimization, Guangxi Engineering Research Center for Pharmaceutical Molecular Screening and Druggability Evaluation, Guilin Medical University, Guilin 541199, China; 3 Department of Pharmacology, School of Pharmacy, Guilin Medical University, Guilin 541199, China; 4 School of Chemistry and Pharmaceutical Sciences, Guangxi Normal University, Key Laboratory for Chemistry and Molecular Engineering of Medicinal Resources (Ministry of Education of China), Guangxi Key Laboratory of Chemistry and Molecular Engineering of Medicinal Resources, Guilin 541004, China; panchx@gxnu.edu.cn; 5 Guangxi Key Laboratory of Enviromental Exposureomics and Entire Lifecycle Health, Guilin 541101, China; 6 Guangxi Colleges and Universities Key Laboratory of Medical Biotechnology and Translational Medicine, Guilin 541199, China; 7 School of Laboratory Medicine and Biotechnology, Guilin Medical University, Guilin 541100, China

**Keywords:** polycystic ovary syndrome, vitexin, nuclear receptor, fibrosis, NLRP3 signaling pathway

## Abstract

**Objective:** In this study, Dehydroepiandrosterone (DHEA-induced Polycystic Ovary Syndrome (PCOS) mice were used as models to evaluate the improvement effect of Vitexin (Vit) on ovarian fibrosis and explore the mechanism of action of the NR4A1/NLRP3 signaling pathway. **Method:** Sixty 4-week-old female ICR mice of the same batch number were selected and their systems were divided into 6 groups (*n* = 10): normal (Control, Ctrl) group, model (Polycystic Ovary Syndrome, PCOS) group, treatment (Vitexin, The Vit group, normal *NR4A1* gene silencing group (Ctrl *NR4A1*^-/-^), *NR4A1* gene silencing model group (PCOS *NR4A1*^-/-^), and *NR4A1* gene silencing treatment group (Vit *NR4A1*^-/-^). Silencing gene modeling was performed by tail vein injection of adeno-associated virus (serotype AAV-8), and the mouse genotypes were detected by qRT-PCR technology 14 days after injection. After the genotype was determined, the PCOS group and the PCOS *NR4A1*^-/-^ group were administered dehydroepandrosterone (6 mg/100 g/d) by gavage for 28 consecutive days for modeling, while the Vit group and the Vit *NR4A1*^-/-^ group were treated with dehydroepandrosterone + vitexin (10 mg/kg/d) by gavage for 28 consecutive days. All mice were raised with pure water and regular maintenance food. After 4 weeks of drug intervention, the mice were euthanized and samples were collected. The pathological changes in ovarian tissue were observed by H&E staining, and the degree of ovarian tissue fibrosis was observed by Masson staining. The levels of superoxide dismutase (SOD), catalase (CAT), glutathione peroxidase (GSH-Px), malondialdehyde (MDA), total cholesterol (TC), triglycerides (TG), high-density lipoprotein cholesterol (HDL-C), and low-density lipoprotein cholesterol (LDL-C) in mouse serum were detected by biochemical kits. The levels of inflammatory factors (IL-1β, IL-6, IL-18, TNF-α) in mouse serum were determined by enzyme-linked immunosorbent assay. Real-time fluorescence quantitative PCR (qRT-PCR) was used to detect oxidative kinase (*Gsta4*, *Prdx3*, *Mgst1*, *Gpx3*, *Gsr*), inflammatory factors (*Nlrp3*, *Caspase-1*, *Asc*, *Il-1β*, *Il-18*, *Tnf-α*) and fibrotic pathway-related genes (*Tgf-β1*, *Smad3*, *Collagen1*, *CTGF*, *α-SMA*, *Mmp-13*, and *β-catenin*) in ovarian tissues. The levels of inflammatory factors (NLRP3, Caspase-1, ASC, IL-1β, IL-18, TNF-α, IκBα) and fibrosis in mice were determined by Western blot method, and statistical description and analysis were performed using SPSS software. **Result:** In the wild-type genotype group, compared with the PCOS group, Vit treatment could effectively regulate the metabolic abnormalities of PCOS mice, including inhibiting excessive weight gain, restoring normal glucose tolerance, and reducing body fat content. After Vit treatment, the levels of MDA, TC, TG, LDL, IL-1β, IL-6, IL-18 and TNF-α in the serum of PCOS mice were significantly reduced, while the levels of SOD and HDL in the serum of PCOS mice were increased. The staining results indicated that Vit treatment could significantly inhibit the process of ovarian fibrosis in PCOS mice. The results of WB and PCR demonstrated that after Vit gavage treatment in mice, inflammatory and fibrotic factors such as Nlrp3, Caspase-1, Asc, Il-1β, Il-18, Tgf-β1, Smad3, Collagen1, CTGF, and α-SMA in ovarian tissues could be significantly down-regulated, and the fibrotic level of ovarian tissues could be reduced. Among the same measurement indicators, the silenced NR4A1 group showed a certain degree of increase compared with the wild genotype group, but there was no significant difference. **Conclusions:** Vit intervention can restore the sex hormone levels and follicular development in ovarian tissues of PCOS mice, regulate reproductive endocrine disorders and abnormal lipid metabolism levels, and regulate the expression of Collagen I, a-SMA and CTGF in the ovaries by inhibiting the NR4A1/NLRP3 signaling pathway, thereby improving the ovarian fibrosis level of PCOS mice. It is suggested that it may play a key role in the treatment of PCOS and the prevention and delay of its long-term complications.

## 1. Introduction

Polycystic Ovary Syndrome (PCOS) is a common reproductive endocrine and metabolic disorder. Its clinical features include ovulatory dysfunction, hyperandrogenemia, and polycystic ovarian morphology [1]. The prevalence of PCOS among women of reproductive age is approximately 6% to 13% worldwide, yet up to 70% of patients remain undiagnosed [2]. The clinical manifestations of PCOS are diverse. These include menstrual irregularities, hirsutism, acne, obesity, and infertility. The etiology of PCOS is not fully understood. However, it is closely associated with genetic, epigenetic, and environmental factors [3]. PCOS not only affects reproductive function but is also frequently accompanied by metabolic disturbances such as insulin resistance, type 2 diabetes, oxidative stress, chronic inflammation, and dyslipidemia. These conditions significantly increase the risk of cardiovascular disease and severely impact patients’ quality of life and long-term health [4]. In both clinical studies and animal experiments, chronic inflammation has been identified as a key pathological feature of PCOS. It interacts with metabolic abnormalities including obesity, insulin resistance, and hyperandrogenemia, collectively driving disease progression [5]. In addition, alterations in the ovarian microenvironment, particularly the development of ovarian fibrosis, may disrupt follicular development and ovulation, further exacerbating the disease process [6]. Therefore, investigating the mechanisms that jointly regulate inflammation and fibrosis in PCOS is of great importance for understanding its pathogenesis and identifying new therapeutic targets.

NR4A1 (nuclear receptor subfamily 4 group A member 1) belongs to the NR4A subfamily within the nuclear receptor superfamily. It is an immediate early response gene that can be rapidly induced by various stimuli including inflammatory cytokines, growth factors, and mechanical stress [7]. Unlike other classical nuclear receptors, NR4A1 does not require exogenous ligands for activation. Its transcriptional activity is primarily regulated by post-translational modifications and subcellular localization [8]. In terms of anti-inflammatory mechanisms, NR4A1 inhibits the p38MAPK/NF-κB signaling pathway and reduces the expression of pro-inflammatory cytokines [9]. For example, in a model of postoperative ileus, activation of NR4A1 significantly suppressed the phosphorylation of p38MAPK and NF-κB, thereby reducing the production of inflammatory mediators such as IL-6 [10]. Moreover, NR4A1 exerts post-transcriptional regulation by directly binding to Tnf mRNA and promoting its degradation, which subsequently downregulates TNF-α expression [11]. NR4A1 is also essential for the maintenance of immune tolerance. Loss of NR4A1 leads to reactivation of the NF-κB signaling pathway and upregulation of pro-inflammatory cytokines [12]. In the context of anti-fibrosis, NR4A1 acts as an endogenous inhibitor of TGF-β signaling [13]. It recruits proteins including SP1, SIN3A, CoREST, LSD1, and HDAC1 to form a transcriptional repressor complex, thereby limiting the pro-fibrotic effects of TGF-β [14]. Notably, in various fibrotic diseases, sustained TGF-β signaling suppresses the expression and functional activity of NR4A1 itself through AKT- and HDAC-dependent pathways, creating a vicious cycle [15]. These lines of evidence indicate that NR4A1 occupies a central position in the regulatory network governing inflammation and fibrosis. Its functional deficiency or abnormal expression may serve as a key driver in a range of inflammation-related diseases.

In recent years, the negative regulatory role of NR4A1 on the NLRP3 inflammasome has been increasingly recognized. The NLRP3 (NOD-like receptor family pyrin domain containing 3) inflammasome is one of the most extensively studied inflammasomes. It is composed of NLRP3, apoptosis-associated speck-like protein containing a CARD (ASC), and caspase-1, and plays a central role in innate immune responses and inflammatory processes [16]. The NLRP3 inflammasome can be activated by various pathogen-associated molecular patterns (PAMPs) and damage-associated molecular patterns (DAMPs). This activation leads to caspase-1 activation, which in turn promotes the maturation and secretion of pro-inflammatory cytokines such as IL-1β and IL-18, and can induce pyroptosis [17]. Studies have shown that NR4A1 inhibits excessive activation of the NLRP3 inflammasome through multiple mechanisms. In macrophages, NR4A1 suppresses the assembly of the NLRP3 inflammasome and the activation of caspase-1, thereby reducing IL-1β secretion [18]. This mechanism may involve the inhibition of the NF-κB signaling pathway by NR4A1, leading to decreased transcriptional expression of NLRP3 and pro-IL-1β. Furthermore, NR4A1 can promote mitophagy, which removes reactive oxygen species (ROS) generated from damaged mitochondria and subsequently blocks NLRP3 inflammasome activation [19]. These findings indicate that NR4A1 serves as an upstream negative regulator of the NLRP3 inflammasome and plays a critical role in the fine-tuning of inflammatory responses.

Under the pathological conditions of PCOS, abnormal activation of the NLRP3 inflammasome has been widely confirmed. The expression levels of NLRP3, caspase-1, and IL-1β are significantly elevated in peripheral blood mononuclear cells and ovarian tissues of PCOS patients. These increases are closely associated with insulin resistance, hyperandrogenemia, and chronic inflammation [20]. Animal studies have further demonstrated that inhibition of NLRP3 inflammasome activity significantly improves ovarian inflammation, insulin resistance, and ovulatory dysfunction in PCOS model mice [21]. Oxidative stress and mitochondrial dysfunction in the ovarian microenvironment are important factors that trigger NLRP3 inflammasome activation [22]. Given the negative regulatory effect of NR4A1 on the NLRP3 inflammasome, and the currently unclear functional status of NR4A1 in the ovarian microenvironment of PCOS, it is hypothesized that NR4A1 may participate in regulating the local ovarian inflammatory response in PCOS by inhibiting NLRP3 inflammasome activation. However, the regulatory role of NR4A1 on the NLRP3 inflammasome in PCOS and the underlying molecular mechanisms remain poorly understood.

Based on this background, identifying exogenous compounds that can effectively regulate the expression or activity of NR4A1 may offer new strategies for the treatment of PCOS. Vitexin is a naturally occurring flavonoid compound. In recent years, it has attracted extensive attention due to its diverse pharmacological activities, including anti-inflammatory, anti-tumor, cardiovascular protective, and neuroprotective effects [23]. With regard to anti-inflammatory activity, vitexin reduces the production of inflammatory mediators by inhibiting the phosphorylation of mitogen-activated protein kinases (MAPKs) such as p38, ERK1/2, and JNK [24]. For example, in a mouse model of Aspergillus fumigatus keratitis, vitexin significantly reduced corneal inflammatory cell infiltration and decreased the expression of inflammatory factors including IL-1β and IL-6 [25]. In terms of neuroprotection, vitexin alleviates cerebral ischemia-hypoxia injury by modulating apoptotic signaling pathways, such as increasing the expression of the anti-apoptotic protein Bcl-2 and inhibiting the expression of the pro-apoptotic protein Bax [26]. Additionally, vitexin can modulate the gut microbiota, ameliorate intestinal inflammation, and reduce the risk of type 2 diabetes [27]. Although vitexin has demonstrated potent anti-inflammatory and tissue-protective effects in various disease models, no studies have yet investigated whether it affects the activation of the NLRP3 inflammasome by regulating NR4A1, thereby interfering with local ovarian inflammation and fibrosis in PCOS.

In summary, this study aims to use vitexin as an interventional agent to explore its regulatory effect on NR4A1 and its subsequent impact on the NLRP3 inflammasome. The goal is to elucidate the potential mechanisms underlying its role in PCOS-associated inflammation and fibrosis, thereby providing a new theoretical basis and experimental foundation for the clinical treatment of PCOS.

## 2. Materials and Methods

### 2.1. Materials

Centrifuge tubes (200 μL, 1.5 mL, 2 mL, 15 mL, 50 mL); Strip tubes (100 μL); Disposable pipette tips (10 μL, 200 μL, 1000 μL); Grinding beads (3 mm); PVDF membrane (purchased from Wuhan Servicebio Technology Co., Ltd., Wuhan, China).

### 2.2. Reagents

Vitexin (Vit, purity > 98%, product code: DST230713-034); RNAi-Easy (AAV8-NR4A1-RNAi; triple protection; Purchase from Shanghai Jikai Gene Technology Co., Ltd., Shanghai, China); Dehydroepiandrosterone (DHEA); Loeffler’s basic methylene blue staining solution (0.23%); Polyethylene glycol 400 (PEG400); Normal saline; Dimethyl sulfoxide (DMSO); Tween 80 (purchased from Shanghai Huayuan Bio-Pharmaceutical Technology Co., Ltd., catalog no. HY-14650, Shanghai, China); TransScript All-in-One RT Super Mix for qPCR (One-Step gDNA Remover) (reverse transcription kit); 2× Universal Blue SYBR Green qPCR Master Mix (qPCR substrate premix); DEPC water; PCR primers (purchased from Wuhan Servicebio Technology Co., Ltd., Wuhan, China); SDS-PAGE gel premix; Ammonium persulfate (APS); TEMED; Recombinant polyclonal primary antibody (anti-rabbit); Recombinant polyclonal secondary antibody (goat anti-rabbit); ECL chemiluminescent substrate (purchased from Beyotime Biotechnology, Shanghai, China).

### 2.3. Animals and Related Materials

ICR mice (purchased from Hunan Slack Jingda, Hunan, China, including quarantine fees, specialized transport boxes, ice packs, transportation, and delivery fees), Standard maintenance diet (purchased from Hunan Slack Jingda), Corn cob bedding (purchased from Hunan Slack Jingda), Drinking water (prepared by boiling water), Disposable blood collection needles, Disposable blood glucose test strips, Multifunctional glucometer (Purchased from Sinocare Inc., Changsha, China). Mice were housed in individually ventilated cages with a population density of 5 per cage. Environmental conditions were controlled as follows: temperature 22 ± 2 °C, relative humidity 50 ± 10%, and a 12 h light/12 h dark cycle (lights turned on at 9:00 AM). The bedding consisted of pine sawdust, and each cage was equipped with nesting materials (cotton blocks) and dried bamboo tube tunnels to meet the behavioral requirements for burrowing and sheltering. Standard maintenance feed and autoclaved drinking water were provided free of charge. Cage bedding was replaced every 3 days.

### 2.4. Instruments

QuantStudio 6 Flex Real-Time PCR System; Pro Flex Gradient PCR System (Provided by Thermo Fisher Scientific Inc.); DM4 B Upright Fluorescence Microscope (Provided by Leica Camera AG, German); Eppendorf Centrifuge (5430R) (Eppendorf AG, German); Constant voltage and current power supply; Automated imaging system (purchased from Wuhan Servicebio Technology Co., Ltd., Wuhan, China).

### 2.5. Animal Modeling and Treatment

Randomized mice were divided into 6 groups using a computer-generated random number sequence (https://www.random.org/, accessed on 15 April 2024), with 10 mice per group. All animals were included in the analysis without exclusion. Sample size was determined through power analysis based on the effect size of ovarian collagen area in preliminary experiments (f = 0.45) (α = 0.05, power = 0.80). During outcome evaluation, laboratory personnel performing biochemical assays, ELISA, qRT-PCR, Western blotting, and histological quantitative analysis remained unaware of the grouping assignments (blinded method). Randomization codes were kept concealed until all data analyses were completed. Forty-eight 4-week-old female wild-type ICR mice were randomly divided into six groups: Control (Ctrl), Polycystic Ovary Syndrome (PCOS), Vitexin Treatment (Vit), NR4A1 Knockdown Control (Ctrl NR4A1^-/-^), NR4A1 Knockdown PCOS (PCOS NR4A1^-/-^), and NR4A1 Knockdown Vitexin Treatment (Vit NR4A1^-/-^). After a 1-week acclimatization period, adeno-associated virus (AAV) was diluted to approximately 5 × 10^12^ v.g./mL and administered via tail vein injection to achieve targeted gene silencing. One week later, the PCOS, Vit, PCOS NR4A1^-/-^, and Vit NR4A1^-/-^ groups underwent drug administration for modeling and treatment.

The modeling agent (DHEA) was completely dissolved in an appropriate volume of DMSO, followed by the sequential addition of PEG400 and Tween80 in a specific ratio. The final volume ratio was DMSO:PEG400:Tween80:NS = 10:30:5:55. After complete dissolution, normal saline was added to dilute the solution to 9 mg/mL. Each mouse in the PCOS, Vit, PCOS NR4A1^-/-^, and Vit NR4A1^-/-^ groups received 0.2 mL of this solution daily via gavage to induce the PCOS mouse model.

The therapeutic agent (Vitexin) was completely dissolved in an appropriate volume of DMSO, followed by the sequential addition of PEG400 and Tween80 in a specific ratio. The final volume ratio was DMSO:PEG400:Tween80:NS = 10:30:5:55. After complete dissolution, normal saline was added to dilute the solution to 6 mg/mL. For mice in the Vit and VitNR4A1-/- groups, the modeling agent (DHEA) and vitexin were administered simultaneously by oral gavage. Each mouse received a total volume of 0.2 mL per administration containing both agents. During the remainder of the experiment, mice were fed a standard diet and provided with normal drinking water [28].

All 60 mice enrolled at the beginning of the experiment were included in the experimental process. Prior to data analysis, predefined criteria for outlier identification were established: values exceeding ±3 standard deviations of the intra-group mean across all indicators were considered severe outliers and excluded. All remaining data after exclusion were incorporated into statistical analysis. No additional data points were excluded [29].

#### 2.5.1. Glucose Tolerance and Body Weight Measurement

During the experimental period, fasting blood glucose and body weight were measured every three days. On the day before measurement, all food was removed from the cages, while water was provided as usual to ensure an 8 h fasting period. On the measurement day, mice were weighed, and their tails were pricked to obtain blood samples for glucose determination using a glucometer. All body weights and fasting blood glucose levels were recorded [30].

#### 2.5.2. Determination of Gene Silencing Efficiency

In the second week after viral injection, 1–2 mm of the tail tip was clipped from mice in the gene silencing groups (Ctrl NR4A1^-/-^, PCOS NR4A1^-/-^, Vit NR4A1^-/-^) and the control group (Ctrl) using sterile, enzymeree instruments. The tail samples were placed in centrifuge tubes containing TRIZOL and grinding beads, homogenized according to a preset program, and total RNA was extracted using chloroform and isopropanol. Reverse transcription and qRT-PCR were performed according to the kit instructions, and the expression levels of NR4A1-related genes were calculated using the 2^−ΔΔCt^ method [31].

#### 2.5.3. Oral Glucose Tolerance Test

On day 28 of the experiment, all mice were fasted for 8 h with free access to water. A glucose solution (0.15 g/mL) was prepared by dissolving a measured amount of glucose in distilled water. Each mouse received 0.2 mL of the glucose solution via gavage. Blood glucose levels were measured at 0, 30, 60, 90, and 120 min, and the oral glucose tolerance was calculated using a standard formula [32].

#### 2.5.4. Animal Observation and Dissection

All mice were monitored twice daily (at 10:00 AM and 4:00 PM) for signs of pain, disease, or unexpected adverse events, including abnormal posture, bristling, lethargy, wound infection, rapid weight loss, or death. humane endpoints were predefined as: (1) weight loss exceeding 20% of initial body weight within 48 h; (2) inability to obtain food or water; (3) persistent hunched posture with severe bristling; (4) near-death status or absence of response to minor stimuli. Any mouse meeting any of these endpoints was immediately euthanized via cervical dislocation under isoflurane anesthesia. No adverse events were observed in this study, and no mice reached humane endpoints prior to scheduled euthanasia on day 28. To minimize potential confounding biases, the following measures were implemented in this study: (1) All mice were derived from the same batch and strain (Hunan Slayke Jingda, Hunan, China), with an initial age of 4 weeks and no significant intergroup differences in baseline body weight; (2) Computerized randomization was employed to ensure balanced baseline body weight and blood glucose levels across groups; (3) All housing conditions (temperature, humidity, lighting, bedding, feed, and water) were maintained consistently; (4) All drug preparation and administration procedures were performed by the same personnel at identical time points; (5) Outcome assessments were conducted using a blinded protocol. Nevertheless, due to the complexity of the PCOS model, certain uncontrolled individual variability factors (e.g., basal metabolic rate, microbiome composition) may be considered as interindividual differences without compromising the validity of conclusions. After 28 days of experimentation, all mice were dissected. Mice were anesthetized via intraperitoneal injection of Avertin, and their body length and weight were measured post-anesthesia. Blood was drawn from the abdominal aorta, allowed to stand at room temperature for 12 h, and then centrifuged to obtain serum, which was stored at −80 °C. The bilateral ovaries, liver, and kidneys were excised and placed in centrifuge tubes containing tissue fixative. Other tissues were placed in centrifuge tubes, snaprozen in liquid nitrogen, and stored at −80 °C. Mouse carcasses and other waste were disposed of according to regulations [33].

#### 2.5.5. Histological Section Staining and Observation

Six ovaries from each group were promptly fixed in 10% neutral buffered formalin for 24–48 h to stabilize protein structures. After fixation, the tissues were dehydrated in a graded ethanol series (70%, 95%, and 100% ethanol) for 1 h each, followed by two 30 min xylene treatments to remove water from the tissues. Subsequently, the tissues were immersed in soft paraffin (52–54 °C) and hard paraffin (56–58 °C) for 1.5 h each in a 60 °C incubator to allow paraffin to fully penetrate the interstitial spaces. The tissue blocks were then placed in embedding cassettes and embedded with optimized paraffin (56–58 °C) before being rapidly cooled and solidified to form paraffin-embedded tissue blocks. The tissue blocks were sectioned into 4 μm-thick continuous sections using a rotary microtome. The sections were floated in a 45 °C water bath to unfold and then adhered to poly-L-lysine-coated slides. Finally, the slides were baked in a 60 °C oven for 2 h to complete the pre-treatment before antigen retrieval.

After baking the paraffin sections at 60 °C for 30 min, they were immersed in xylene I and II for 10 min each to deparaffinize, followed by rehydration through a graded ethanol series (100% → 95% → 85% → 75% ethanol) to distilled water. The nuclei were stained with Weigert’s iron hematoxylin for 8 min, then blued in tap water and differentiated in a 1% phosphomolybdic acid solution for 5 min. Subsequently, the muscle fibers and cytoplasm were stained with an acid fuchsin-picroindigocarmine mixture for 5 min, followed by differentiation in a 2% phosphomolybdic acid solution until the collagen appeared light pink and the muscle fibers appeared red. The sections were immediately counterstained with a 1% light green solution for 3 min and rinsed in 0.2% glacial acetic acid to terminate the reaction. The sections were then dehydrated through a graded ethanol series (75% → 85% → 95% → 100% ethanol for 30 s each), cleared in xylene for 2 × 3 min, and mounted with neutral gum. The final Masson’s trichrome-stained sections displayed high-contrast images with bright green collagen fibers, red muscle fibers and red blood cells, and blue-black nuclei. The area of collagen fibers was quantitatively measured using ImageJ (1.54m 5 December 2024) software, collagen area quantification was performed by two independent investigators blinded to group allocation; inter-rater reliability (ICC) was 0.87 (95% CI: 0.82–0.92), indicating excellent agreement [34].

#### 2.5.6. ELISA and Biochemical Assays Using Mouse Serum

Mouse serum was used to measure levels of sex hormones (AMH, FSH, LH, T, E2), lipid profile (TG, TC, HDL-C, LDL-C), and inflammatory markers (TNF-α, IL-1β, IL-6, IL-18) using ELISA and biochemical kits. Absorbance was measured using a microplate reader [35].

#### 2.5.7. qRT-PCR Experiment

Ovaries from both sides of the mice were placed in centrifuge tubes containing TRIZOL and grinding beads and thoroughly homogenized at 4 °C. Total RNA was extracted using chloroform and isopropanol. After measuring RNA concentration using a microvolume nucleic acid protein analyzer, the concentration was adjusted appropriately. Reverse transcription was performed using a reverse transcription kit. Target primers and corresponding substrates were added and measured on a real-time PCR analyzer. Target genes included inflammatory factors (Caspase-1, IL-1β, IL-18, NLRP3, ASC, Iκbα, TNF-α) and fibrosis markers (α-SMA, smad3, CTGF, Collagen1, MMP-13) Table 1 [36].

#### 2.5.8. Western Blot Experiment

Approximately 20 mg of mouse ovaries were taken, residual blood was removed, and RIPA lysis buffer and grinding beads were added. The tissues were thoroughly homogenized at 4 °C and incubated on ice for 1 h to fully lyse. After centrifugation, the supernatant was collected, and protein concentration was determined using a BCA kit. After incubation, absorbance was measured at 562 nm to calculate protein concentration. Based on the calculated concentration, an appropriate amount of PBS and loading buffer was added, mixed thoroughly, and heated at 95 °C for 5 min. After cooling, the samples were subjected to Western blot analysis.

SDS-PAGE gel premix was prepared by adding APS and TEMED, quickly mixed, and poured into a pre-leak-tested gel casting glass plate. After solidification, the top layer gel was prepared in the same manner, poured into the glass plate, and a 15-well comb was quickly inserted. After the top layer gel solidified, the gel was placed in the electrophoresis apparatus. Electrophoresis buffer was prepared at a certain ratio and poured into the electrophoresis apparatus and tank. The constant voltage power supply was connected and set to 80 V to start electrophoresis. After a certain period, the power was turned off. The gel was removed, and a PVDF membrane activated with methanol was placed on the gel. The PVDF membrane and protein gel were clamped in the following order: plate, electrotransfer sponge, electrotransfer filter paper, PVDF membrane, gel, electrotransfer filter paper, electrotransfer sponge, plate. The power supply was connected, and the membrane was transferred at 240 mV for 90 min. After transfer, the power was turned off, and the membrane was blocked with skim milk powder at 60 r/min for 2 h. After blocking, the membrane was washed with TBST solution at 120 r/min for 10 min, three times. After washing, the membrane was incubated with corresponding primary antibodies overnight at 4 °C and 60 r/min. After overnight incubation, the primary antibody was recovered, and the membrane was washed as described above. Secondary antibody was added and incubated at room temperature at 60 r/min for 1 h. After incubation, the membrane was washed again. ECL chemiluminescent substrate was evenly applied to the membrane, which was then placed in a chemiluminescent imager for exposure to obtain protein images.

### 2.6. Experimental Data Processing

Data were expressed as mean ± standard deviation. Statistical analysis was performed using SPSS 26.0. Multiple group comparisons were conducted using one-way ANOVA, with post hoc pairwise comparisons performed using Tukey’s HSD test. If variances were not homogeneous (Levene test, *p* < 0.05), Welch ANOVA and Games-Howell post hoc test were employed. Outliers were identified using box plot analysis(Tukey’s test), with values exceeding mean ± 3 standard deviations considered as significant outliers and excluded. Normality was assessed using Shapiro–Wilk test. A *p*-value < 0.05 was considered statistically significant. Each experiment was independently repeated three times, with biological replicates *n* = 6 per group. For primary outcome measures, in addition to *p*-values, the effect size and its 95% confidence interval are reported. The specific types of effect sizes are as follows: for between-group mean comparisons, mean difference (MD) and its 95% CI are used; for categorical variables, rate difference or odds ratio is employed; in analysis of variance, partial η^2^ is reported as the effect size. All confidence intervals are presented at a two-sided 95% level.

## 3. Results

### 3.1. Changes in Body Weight

**Figure 1:**
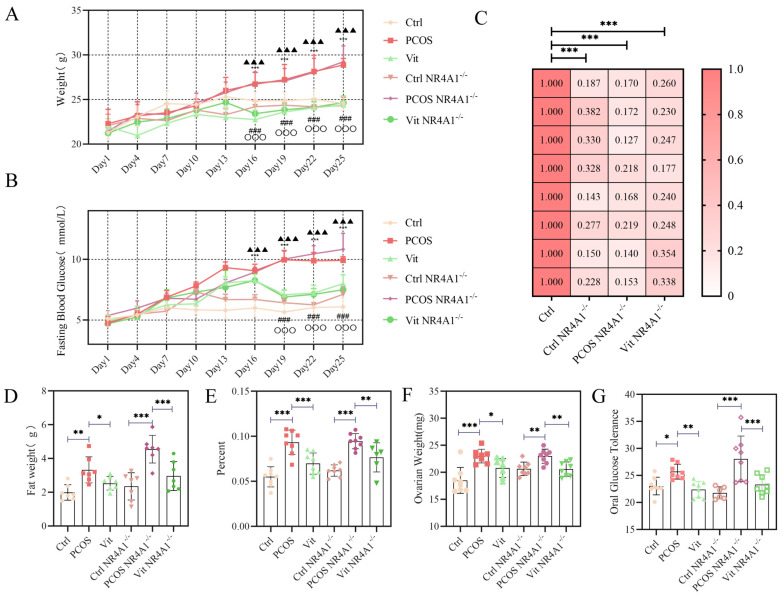
In Vivo Data: (**A**): Fasting Body Weight Data; (**B**): Fasting Blood Glucose Data; (**C**): NR4A1^-/-^ Gene Silencing Data; (**D**): Fat Weight Data; (**E**): Ovarian Index Data; (**F**): Ovarian Weight Data; (**G**): Oral Glucose Tolerance Data; Biological Replicates (*n* = 6); Experimental Repetition (*n* = 3); Symbol explanation: Figure (**A**): #: Ctrl vs. PCOS group; *: PCOS group vs. Vit group; ▲: Ctrl *NR4A1*^-/-^ vs. PCOS *NR4A1*^-/-^ group; ◌: PCOS *NR4A1*^-/-^ vs. Vit *NR4A1*^-/-^ group. Significance Levels: *n* = 1, * *p* < 0.05; *n* = 2, ** *p* < 0.01; *n* = 3, *** *p* < 0.001. All data are presented as mean ± standard error of the mean (mean ± SD).

As shown in Figure 1A, starting from day 16, the fasting body weight of the PCOS group began to show a significant difference compared to the Ctrl and Vit groups (*p* < 0.05). The PCOS NR4A1^-/-^ group exhibited a significant difference in fasting body weight compared to the Ctrl NR4A1^-/-^ and Vit NR4A1^-/-^ groups on day 22 (*p* < 0.05). From the start of the experiment to day 13, all groups showed varying degrees of weight increase. Thereafter, the body weight of the Ctrl group gradually stabilized. Significant differences were observed between the Ctrl and Vit groups on days 13 and 16 (*p* < 0.05). Subsequently, the body weight of the Vit group began to increase again, eventually reaching a level similar to that of the Ctrl group. The overall trends of the Ctrl NR4A1^-/-^ and Vit NR4A1^-/-^ groups were consistent with those of the Ctrl and Vit groups, with no significant differences observed between groups (*p* > 0.05). These results indicate a clear trend of increased body weight in PCOS and NR4A1-silenced PCOS mice, which is consistent with clinical manifestations.

### 3.2. Changes in Fasting Blood Glucose

As shown in Figure 1B, starting from day 13, the fasting blood glucose levels of the PCOS and PCOS NR4A1^-/-^ groups exceeded 8 mmol/L, meeting the criteria for hyperglycemia according to relevant animal experimental standards. The fasting blood glucose of the Vit group peaked on day 10 and then stabilized at a lower level. The PCOS and PCOS NR4A1^-/-^ groups exhibited a steady increase in blood glucose levels after the start of the experiment, showing significant differences compared to the Vit and Vit NR4A1^-/-^ groups on day 19 (*p* < 0.05) and compared to the Ctrl and Ctrl NR4A1^-/-^ groups on day 16 (*p* < 0.05). The trends in the Ctrl NR4A1^-/-^ and Vit NR4A1^-/-^ groups were similar to those of the Ctrl and Vit groups, with no significant differences observed on a daily basis (*p* > 0.05). These results indicate that PCOS causes an increase in fasting blood glucose levels, which is consistent with relevant animal experimental standards and clinical research evidence.

### 3.3. Efficiency of Gene Silencing

As shown in Figure 1C, the relative expression levels of NR4A1 were calculated using Ct values and normalized with the 2^−ΔΔCt^ method. Compared to the Ctrl group, the relative expression levels of NR4A1 in the Ctrl NR4A1^-/-^, PCOS NR4A1^-/-^, and Vit NR4A1^-/-^ groups were 27.31%, 18.11%, and 23.54%, respectively. One-way ANOVA analysis revealed significant differences between the relative expression levels of the NR4A1-silenced groups and the Ctrl group (*p* < 0.05). These findings demonstrate that the adenovirus-associated virus-mediated gene silencing achieved the experimental objectives in terms of efficiency and stability, allowing for subsequent experiments.

### 3.4. Fat Weight Ratio

As shown in Figure 1D, bilateral inguinal subcutaneous adipose tissue was rapidly dissected and weighed after euthanasia on day 28 of the experiment, with careful removal of fascia and blood vessels. The results demonstrated that the inguinal fat weight in the Ctrl group was approximately 3.5 g, whereas the PCOS group exhibited a significant increase to approximately 6.5 g, representing an 85.7% elevation compared to the Ctrl group (*p* < 0.001), indicating pronounced pathological accumulation of subcutaneous adipose tissue under PCOS pathological conditions. The Vit group showed a significant reduction to approximately 4.5 g, representing a 30.8% decrease compared to the PCOS group (*p* < 0.01), although remaining slightly elevated relative to the Ctrl group, suggesting that Vitexin intervention effectively alleviated PCOS-associated adipose tissue hyperplasia. The Ctrl NR4A1-/- group exhibited approximately 4.5 g, with no significant difference compared to the Ctrl group (*p* > 0.05), implying that NR4A1 gene silencing per se has limited impact on adipose metabolism in normal mice. Notably, the PCOS NR4A1-/- group demonstrated a further increase to approximately 7.5 g, representing a 15.4% elevation compared to the PCOS group (*p* < 0.001) and a 66.7% increase compared to the Ctrl NR4A1-/- group (*p* < 0.001), indicating that NR4A1 gene silencing significantly exacerbated PCOS-associated abnormal adipose tissue accumulation. The Vit NR4A1-/- group exhibited approximately 5.5 g, representing a significant 26.7% reduction compared to the PCOS NR4A1-/- group (*p* < 0.001), yet remained significantly elevated compared to the Ctrl NR4A1-/- group (*p* < 0.05), suggesting that the ameliorative effect of Vitexin on inguinal adipose tissue was markedly attenuated under NR4A1 gene silencing conditions, failing to restore to normal levels. Collectively, these findings indicate that PCOS induces significant augmentation of inguinal subcutaneous adipose tissue, which is further aggravated by NR4A1 gene silencing, whereas Vitexin exerts lipid-lowering effects potentially through activation of NR4A1-associated signaling pathways, implicating NR4A1 as a critical molecular target for Vitexin in ameliorating metabolic disturbances associated with PCOS.

### 3.5. Ovarian Weight and Ovarian Index

As depicted in Figure 1E,F, the ovaries from both sides of the mice were excised post-mortem, with surrounding fat and residual blood removed before weighing. Compared to the Ctrl and Ctrl NR4A1^-/-^ groups, the PCOS and PCOS NR4A1^-/-^ groups exhibited a significant increase in bilateral ovarian weight (*p* < 0.05), indicating that the ovaries in these groups were affected by endocrine abnormalities in the PCOS environment. Conversely, the ovarian index in the PCOS and PCOS NR4A1^-/-^ groups showed a marked decline compared to the Ctrl and Ctrl NR4A1^-/-^ groups, suggesting that these groups had an excessive accumulation of adipose tissue, leading to abnormal weight gain and suppression of normal ovarian growth and development. In the Vit and Vit NR4A1^-/-^ groups, treatment with Vitexin mitigated the abnormal weight gain and alleviated the impact of the aberrant hormonal environment on the ovaries.

### 3.6. Oral Glucose Tolerance Test

As shown in Figure 1G, oral glucose tolerance was assessed by calculating the area under the curve (AUC) of blood glucose levels during the oral glucose tolerance test (OGTT) on day 28 of the experiment. The results demonstrated that the AUC in the Ctrl group was approximately 23 mmol·h/L, whereas the PCOS group exhibited a significant increase to approximately 26 mmol·h/L, representing a 13.0% elevation compared to the Ctrl group (*p* < 0.05), indicating impaired glucose tolerance under PCOS pathological conditions. The Vit group showed a significant reduction to approximately 22.5 mmol·h/L, representing a 13.5% decrease compared to the PCOS group (*p* < 0.01), and even slightly lower than the Ctrl group, suggesting that Vitexin intervention effectively improved glucose metabolic disturbances and enhanced insulin sensitivity in PCOS mice. The Ctrl NR4A1-/- group exhibited approximately 21.5 mmol·h/L, with no significant difference compared to the Ctrl group (*p* > 0.05), implying that NR4A1 gene silencing per se has limited impact on glucose metabolism in normal mice. Notably, the PCOS NR4A1-/- group demonstrated a marked increase to approximately 29 mmol·h/L, representing an 11.5% elevation compared to the PCOS group (*p* < 0.01) and a 34.9% increase compared to the Ctrl NR4A1-/- group (*p* < 0.01), indicating that NR4A1 gene silencing significantly exacerbated PCOS-associated glucose intolerance and insulin resistance. The Vit NR4A1-/- group exhibited approximately 23.5 mmol·h/L, representing a significant 19.0% reduction compared to the PCOS NR4A1-/- group (*p* < 0.01), yet remained slightly elevated compared to the Ctrl NR4A1-/- group, suggesting that the ameliorative effect of Vitexin on glucose tolerance was partially preserved but attenuated under NR4A1 gene silencing conditions. Collectively, these findings indicate that PCOS induces significant impairment of oral glucose tolerance, which is further aggravated by NR4A1 gene silencing, whereas Vitexin exerts glucose-lowering and insulin-sensitizing effects potentially through activation of NR4A1-associated signaling pathways, implicating NR4A1 as a critical molecular mediator for Vitexin in improving glucose metabolic homeostasis in PCOS.

### 3.7. Histological Analyses

**Figure 2:**
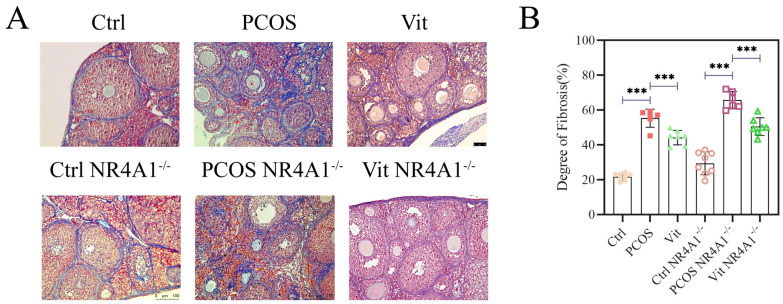
Histological and Fibrosis Data. (**A**): Masson’s trichrome-stained ovarian sections of six groups of mice; (**B**): Comparison of fibrosis degree. Biological Replicates (*n* = 6); Experimental Repetition (*n* = 3); Significance Levels: *** *p* < 0.001. All data are presented as mean ± standard error of the mean (mean ± SD).

As illustrated in the Figure 2, the average area percentage of collagen fibers in the ovarian tissues of each group was measured. The results indicated that the average area percentage of collagen fibers in the ovaries of the PCOS and PCOS NR4A1^-/-^ groups was significantly higher than that in the Ctrl and Ctrl NR4A1^-/-^ groups (*p* < 0.05). In contrast, the Vit and Vit NR4A1^-/-^ groups exhibited a lower collagen fiber area percentage compared to the PCOS and PCOS NR4A1^-/-^ groups (*p* < 0.05). These findings suggest that the experimental treatment induced collagen fiber proliferation, leading to an increase in collagen content within the tissues, which was inhibited by Vitexin treatment.

### 3.8. ELISA and Biochemical Kit Analysis

**Figure 3:**
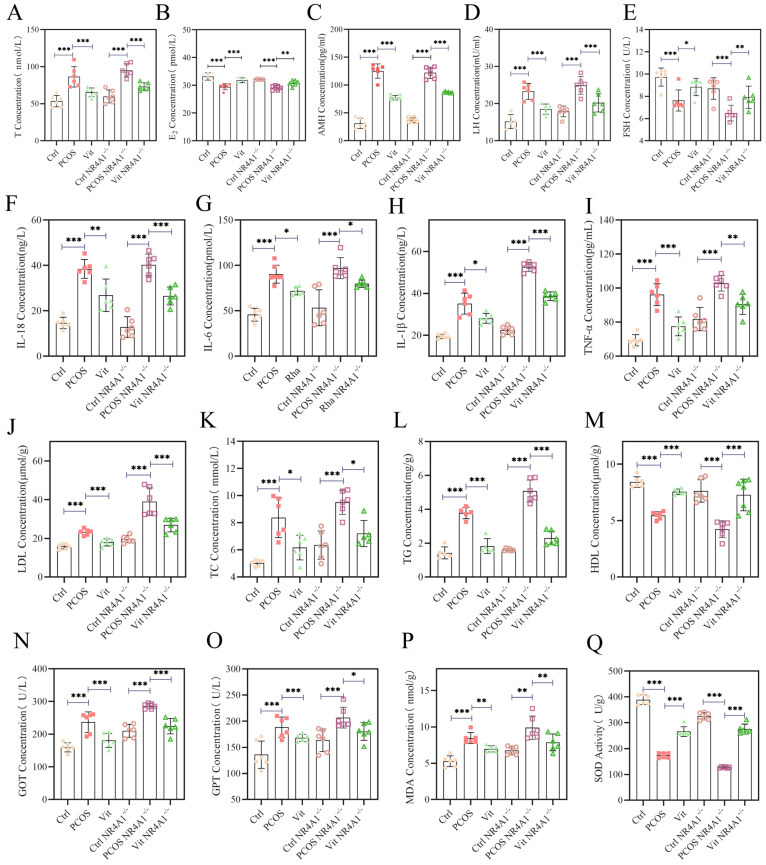
Hormonal and Metabolic Profiles. (**A**): Testosterone (T) Levels; (**B**): Estradiol Hormone (E) Levels; (**C**): Anti-Müllerian Hormone (AMH) Levels; (**D**): Luteinizing Hormone (LH) Levels; (**E**): Follicle-Stimulating Hormone (FSH) Levels; (**F**): Interleukin 18 (IL-18) Levels; (**G**): Interleukin 6 (IL-6) Levels; (**H**): Interleukin 1β (IL-1β) Level;s (**I**): Tumor Necrosis Factor-α (TNF-α) Levels; (**J**): Low-Density Lipoprotein Cholesterol (LDL-C) Levels; (**K**): Total Cholesterol (TC) Levels; (**L**): Triglyceride (TG) Levels; (**M**): High-Density Lipoprotein Cholesterol (HDL-C) Levels; (**N**): Glutamic-Oxaloacetic Transaminase (GOT) Levels; (**O**): Glutamic-Pyruvic Transaminase (GPT) Levels; (**P**): Malondialdehyde (MDA) Levels; (**Q**): Superoxide Dismutase (SOD) Biological Replicates (*n* = 6); Experimental Repetition (*n* = 3); Significance Levels: * *p* < 0.05, ** *p* < 0.01, *** *p* < 0.001. All data are presented as mean ± standard error of the mean (mean ± SD).

As shown in Figure 3, In terms of testosterone (T), the PCOS and PCOS NR4A1-/- groups exhibited a significant increase compared to the Ctrl and Ctrl NR4A1-/- groups (*p* < 0.001). Specifically, serum T concentration in the PCOS group was approximately 95 nmol/L, representing a 58.3% increase compared to the Ctrl group (approximately 60 nmol/L), while the PCOS NR4A1-/- group showed approximately 90 nmol/L, representing a 63.6% increase compared to the Ctrl NR4A1-/- group (approximately 55 nmol/L). The Vit and Vit NR4A1-/- groups demonstrated a significant regulatory effect compared to the PCOS and PCOS NR4A1-/- groups (*p* < 0.001), with T levels decreasing to approximately 75 nmol/L and 70 nmol/L respectively, indicating that Vitexin treatment could effectively suppress hyperandrogenism, although complete normalization was not achieved. In terms of luteinizing hormone (LH), the PCOS and PCOS NR4A1-/- groups exhibited a significant increase compared to the Ctrl and Ctrl NR4A1-/- groups (*p* < 0.001). The Vit and Vit NR4A1-/- groups demonstrated a significant regulatory effect compared to the PCOS and PCOS NR4A1-/- groups (*p* < 0.001), indicating that the PCOS groups had a higher LH production than their respective controls, which was normalized by Vitexin treatment. In the case of follicle-stimulating hormone (FSH), the PCOS and PCOS NR4A1-/- groups also showed a significant increase compared to the Ctrl and Ctrl NR4A1-/- groups (*p* < 0.05). The Vit and Vit NR4A1-/- groups exhibited a significant regulatory effect (*p* < 0.05), suggesting that Vitexin treatment could normalize FSH levels. These results reflect the endocrine imbalances characteristic of PCOS, which were mitigated by Vitexin treatment. Similar trends were observed in anti-Müllerian hormone (AMH) testing. In estradiol (E2) testing, the PCOS and PCOS NR4A1-/- groups exhibited a significant decrease compared to the Ctrl and Ctrl NR4A1-/- groups (*p* < 0.05), while the Vit and Vit NR4A1-/- groups showed a significant recovery (*p* < 0.05). These findings indicate that Vitexin can restore the endocrine imbalances associated with PCOS.

As shown in Figure 3, Low-density lipoprotein cholesterol (LDL-C) levels (Figure 3J) were significantly elevated in the PCOS group compared to the Ctrl group (*p* < 0.001), and further exacerbated in the PCOS NR4A1-/- group relative to the Ctrl NR4A1-/- group (*p* < 0.001), with the PCOS NR4A1-/- group showing the highest LDL-C concentration among all groups. The Vit and Vit NR4A1-/- groups demonstrated significant reductions in LDL-C levels compared to their respective PCOS model groups (*p* < 0.01), though complete normalization was not achieved in the Vit NR4A1-/- group.

Total cholesterol (TC) levels (Figure 3K) followed a similar pattern, with significant increases observed in the PCOS and PCOS NR4A1-/- groups compared to their respective controls (*p* < 0.001). Notably, the PCOS NR4A1-/- group exhibited the most pronounced hypercholesterolemia. Vitexin treatment significantly attenuated TC elevation in both the Vit and Vit NR4A1-/- groups (*p* < 0.05), suggesting a cholesterol-lowering effect independent of NR4A1 status.

Triglyceride (TG) levels (Figure 3L) were markedly elevated in the PCOS group compared to the Ctrl group (*p* < 0.001), with the PCOS NR4A1-/- group showing the most severe hypertriglyceridemia, approximately 3-fold higher than the Ctrl NR4A1-/- group (*p* < 0.001). The Vit and Vit NR4A1-/- groups exhibited significant reductions in TG levels (*p* < 0.001), though the Vit NR4A1-/- group remained significantly elevated compared to its control counterpart.

High-density lipoprotein cholesterol (HDL-C) levels (Figure 3M) presented an inverse pattern, with significant reductions in the PCOS and PCOS NR4A1-/- groups compared to controls (*p* < 0.001), indicating impaired reverse cholesterol transport. Vitexin treatment significantly restored HDL-C levels in the Vit group (*p* < 0.001), with a similar trend observed in the Vit NR4A1-/- group, though the restorative effect was attenuated under NR4A1 silencing conditions.

Glutamate oxaloacetate transaminase (GOT) levels (Figure 3N), a marker of hepatocellular integrity, were significantly elevated in the PCOS and PCOS NR4A1-/- groups compared to controls (*p* < 0.01), with the PCOS NR4A1-/- group showing the highest GOT activity, suggesting NR4A1 silencing exacerbates hepatic injury in PCOS. Vitexin treatment significantly reduced GOT levels in both intervention groups (*p* < 0.05), indicating hepatoprotective effects.

Glutamate pyruvate transaminase (GPT) levels (Figure 3O) demonstrated comparable trends, with significant elevations in PCOS model groups (*p* < 0.05) and significant reductions following Vitexin administration (*p* < 0.05), further supporting the hepatic protective capacity of Vitexin.

Malondialdehyde (MDA) levels (Figure 3P), a direct indicator of lipid peroxidation and oxidative stress, were significantly increased in the PCOS group compared to the Ctrl group (*p* < 0.001), with the PCOS NR4A1-/- group exhibiting the most severe oxidative damage. The Vit and Vit NR4A1-/- groups demonstrated significant reductions in MDA levels (*p* < 0.01), though the Vit NR4A1-/- group failed to reach control levels, suggesting NR4A1 involvement in Vitexin-mediated antioxidant defense.

Superoxide dismutase (SOD) activity (Figure 3Q), a critical enzymatic antioxidant defense mechanism, was significantly suppressed in the PCOS group compared to the Ctrl group (*p* < 0.001), with the PCOS NR4A1-/- group showing the most pronounced reduction in antioxidant capacity. Vitexin treatment significantly restored SOD activity in the Vit group (*p* < 0.01), with a similar but attenuated effect observed in the Vit NR4A1-/- group.

As shown in Figure 3, in tumor necrosis factor-alpha (TNF-α), the PCOS and PCOS NR4A1^-/-^ groups exhibited a significant increase compared to the Ctrl and Ctrl NR4A1^-/-^ groups (*p* < 0.05), indicating a pronounced inflammatory response in the model groups. The Vit and Vit NR4A1^-/-^ groups demonstrated a significant anti-inflammatory effect (*p* < 0.05). In genotype-based comparisons, the TNF-α levels in the NR4A1-silenced groups were somewhat lower than those in the normal genotype groups, possibly due to the multifaceted regulatory effects of NR4A1 on inflammation-related pathways. Similar trends were observed in interleukin-1 beta (IL-1β) and IL-18 testing, with the PCOS and PCOS NR4A1^-/-^ groups showing significant increases compared to the Ctrl and Ctrl NR4A1^-/-^ groups (*p* < 0.05). The Vit and Vit NR4A1^-/-^ groups exhibited significant anti-inflammatory effects (*p* < 0.05). Notably, the PCOS NR4A1^-/-^ group showed a particularly pronounced increase in IL-1β, likely due to the impact of NR4A1 silencing on the expression and secretion of related proteins. The results for IL-6 were consistent with those for the aforementioned cytokines.

### 3.9. qRT-PCR Analysis

**Figure 4:**
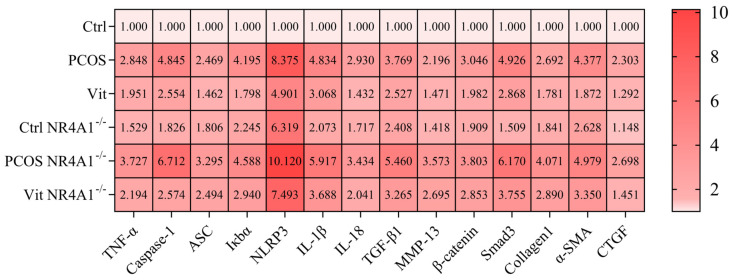
Molecular and Pathway Analysis.

As shown in Figure 4, compared to the Ctrl and Ctrl NR4A1^-/-^ groups, the PCOS and PCOS NR4A1^-/-^ groups exhibited significant upregulation in the gene expression of ASC, caspase-1, NLRP3, IκBα, IL-1β, and IL-18 (*p* < 0.05), indicating activation of the inflammatory signaling pathway and a pronounced inflammatory response. In contrast, the Vit and Vit NR4A1^-/-^ groups showed downregulation of these genes compared to the PCOS and PCOS NR4A1^-/-^ groups (*p* < 0.05). These findings suggest that the inflammatory signaling pathway was activated in PCOS mice, leading to a significant inflammatory response. Similar expression trends were observed for α-smooth muscle actin (α-SMA), connective tissue growth factor (CTGF), and Smad3, reflecting the activation of fibrotic signaling pathways downstream of the inflammatory response, resulting in irreversible fibrotic changes.

### 3.10. Western Blot Analysis

**Figure 5:**
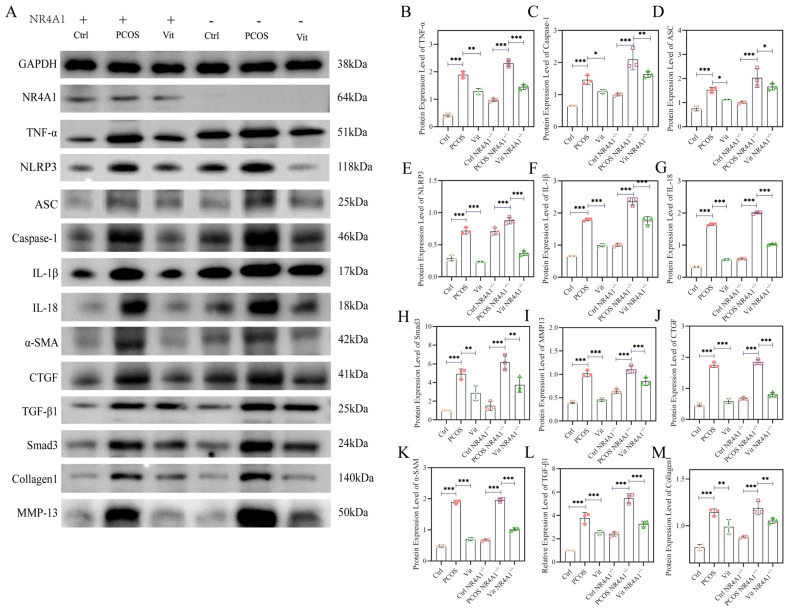
Western Blot Analysis. (**A**): representative Western blot images; (**B**): TNF-α protein expression; (**C**): Caspase-1 protein expression; (**D**): ASC protein expression; (**E**): NLRP3 protein expression; (**F**): IL-1β protein expression; (**G**): IL-18 protein expression; (**H**): TGF-β1 protein expression; (**I**): Collagen1 protein expression; (**J**): Smad3 protein expression; (**K**): α-SMA protein expression; (**L**): MMP-13 protein expression; (**M**): CTGF protein expression; Biological Replicates (*n* = 6); Experimental Repetition (*n* = 3); Significance Levels: * *p* < 0.05, ** *p* < 0.01, *** *p* < 0.001. All data are presented as mean ± standard error of the mean (mean ± SD).

As shown in Figure 5, compared to the Ctrl and Ctrl NR4A1^-/-^ groups, the PCOS and PCOS NR4A1^-/-^ groups exhibited significant upregulation in the relative protein expression of ASC, Caspase-1, IκBα, IL-1β, and IL-18 (*p* < 0.05), indicating activation of the inflammatory signaling pathway and a pronounced inflammatory response. In contrast, the Vit and Vit NR4A1^-/-^ groups showed downregulation of these proteins compared to the PCOS and PCOS NR4A1^-/-^ groups (*p* < 0.05). These findings suggest that Vitexin treatment inhibited the inflammatory response at the protein level. Similar expression trends were observed for TGF-beta1, Collagen1, α-SMA, CTGF, and Smad3, indicating that Vitexin treatment could mitigate fibrosis mediated by the inflammatory response, achieving therapeutic effects in PCOS mice.

## 4. Discussion

### 4.1. Summary of Major Findings

This study investigated whether vitexin ameliorates ovarian fibrosis in PCOS mice through the NR4A1/NLRP3 signaling pathway. The major findings are as follows. First, vitexin treatment significantly reduced body weight gain, fasting blood glucose, fat weight, and oral glucose tolerance test area under the curve in DHEA-induced PCOS mice, indicating improved metabolic abnormalities. Second, Masson’s trichrome staining revealed that vitexin decreased ovarian collagen deposition, while qRT-PCR and Western blotting showed downregulation of fibrosis markers including α-SMA, CTGF, and Collagen1. Third, vitexin suppressed NLRP3 inflammasome activation, as evidenced by reduced expression of NLRP3, ASC, Caspase-1, IL-1β, and IL-18 at both mRNA and protein levels. Fourth, NR4A1 expression was downregulated in PCOS mouse ovaries, and vitexin intervention restored its expression. Notably, in NR4A1-silenced mice (Ctrl NR4A1^-/-^, PCOS NR4A1^-/-^, and Vit NR4A1^-/-^ groups), the protective effects of vitexin against ovarian fibrosis and inflammation were partially attenuated, suggesting that its anti-fibrotic action is at least partially dependent on NR4A1 activation. Collectively, these results demonstrate that vitexin ameliorates PCOS-associated ovarian fibrosis via regulation of the NR4A1/NLRP3 signaling axis.

### 4.2. Vitexin Improves Metabolic Abnormalities and Ovarian Fibrosis in PCOS

The metabolic improvements observed in this study align with the known pharmacological activities of vitexin. Vitexin (apigenin-8-C-β-D-glucopyranoside) is a naturally occurring flavonoid widely distributed in medicinal plants such as Crataegus and Vitex species, with well-documented anti-inflammatory and antioxidant properties [37]. Mechanistically, vitexin inhibits MAPK signaling pathway phosphorylation, reducing pro-inflammatory cytokine production [38], while activating SOD and GSH-Px to attenuate oxidative stress [39]. In the present study, PCOS mice exhibited significant weight gain, hyperglycemia, dyslipidemia (elevated TC, TG, LDL-C and reduced HDL-C), and impaired glucose tolerance—all of which were ameliorated by vitexin intervention. These findings are consistent with previous reports that vitexin improves metabolic disturbances in various disease models [40]. Importantly, the close association between metabolic dysfunction and ovarian fibrosis in PCOS has been increasingly recognized [41]. Chronic low-grade inflammation and oxidative stress, which are hallmarks of PCOS, create a permissive microenvironment for fibroblast activation and extracellular matrix accumulation [42]. Our observation that vitexin simultaneously improved metabolic parameters and reduced ovarian collagen deposition supports the notion that its anti-fibrotic effects may be mediated, at least in part, through metabolic regulation. However, the fact that vitexin directly suppressed fibrotic gene expression in ovarian tissue suggests additional tissue-specific mechanisms.

### 4.3. The NR4A1/NLRP3 Signaling Axis in PCOS-Associated Ovarian Fibrosis

A central finding of this study is the identification of NR4A1 as a key molecular target of vitexin in PCOS-associated ovarian fibrosis. NR4A1 (nuclear receptor subfamily 4 group A member 1) is an orphan nuclear receptor that functions as an immediate-early gene product and serves as a contextual regulator of immunity, metabolism, and stress responses [43]. In the present study, NR4A1 expression was significantly downregulated in ovarian tissues of PCOS mice compared to controls. This observation is consistent with recent findings that NR4A1 expression is reduced in PCOS patients and animal models, and that its downregulation is associated with increased inflammatory and metabolic disturbances [44]. Notably, a recent study identified NR4A1 as a diagnostic biomarker for PCOS, with robust discriminatory capacity [45].

The NLRP3 inflammasome is a key effector of innate immunity that has been implicated in the pathogenesis of PCOS [46]. Upon activation, NLRP3 recruits ASC and pro-caspase-1 to form a multiprotein complex, leading to caspase-1 activation and subsequent maturation of IL-1β and IL-18 [47]. In PCOS patients, elevated NLRP3 inflammasome activity in peripheral blood mononuclear cells correlates with insulin resistance and hyperandrogenemia [46]. Our results demonstrate that in PCOS mouse ovaries, NLRP3 inflammasome components and downstream cytokines are significantly upregulated, and vitexin intervention effectively suppresses this activation. Importantly, we found that NR4A1 silencing partially abolished the inhibitory effect of vitexin on NLRP3 inflammasome activation, indicating that vitexin suppresses NLRP3 at least partially through NR4A1. This finding is consistent with recent in vitro evidence demonstrating that vitexin alleviates DHT-induced fibrosis in KGN cells by activating NR4A1 and inhibiting NLRP3 inflammasome-mediated inflammation [9].

The mechanistic link between NR4A1 and NLRP3 may involve multiple pathways. First, NR4A1 can inhibit NF-κB signaling, which is required for NLRP3 and pro-IL-1β transcription [48]. Second, NR4A1 promotes mitophagy, thereby removing mitochondrial reactive oxygen species that serve as NLRP3 activators [11]. Third, recent evidence suggests that NR4A1 can directly interact with NLRP3 to prevent inflammasome assembly [48]. Moreover, NR4A1 has been shown to recruit histone deacetylases to suppress TGF-β-induced fibrotic gene expression [49], providing an additional layer of anti-fibrotic regulation. Collectively, these findings position NR4A1 as an upstream negative regulator of NLRP3 inflammasome and a critical mediator of vitexin’s protective effects in PCOS. Given that TGF-β signaling is a central driver of ovarian fibrosis [41], the ability of vitexin to counteract this pathway via NR4A1 further underscores its therapeutic potential.

### 4.4. Unexpected Findings and Their Implications

Several unexpected observations merit discussion. First, in NR4A1-silenced groups, certain inflammatory cytokines (particularly TNF-α) showed slightly lower levels compared to wild-type PCOS mice, despite NR4A1 being considered an anti-inflammatory regulator. This seemingly paradoxical finding may be explained by the cell-type-specific and context-dependent functions of NR4A1. While NR4A1 generally suppresses inflammation in macrophages and dendritic cells, it can promote pro-inflammatory responses in certain contexts, such as in T cells where it regulates Th17 differentiation [50]. Alternatively, NR4A1 silencing may trigger compensatory upregulation of other NR4A family members (NR4A2 and NR4A3), which share overlapping functions [51]. The residual NR4A1 expression (approximately 23–27% of control levels) in our knockdown model represents a partial loss-of-function rather than complete ablation, which may also contribute to the observed complexity. Second, we noted that vitexin still exerted partial protective effects in NR4A1-silenced mice, suggesting that NR4A1-independent pathways may also contribute to its anti-fibrotic activity. This is plausible given that vitexin has multiple reported targets, including MAPKs and NF-κB [52]. Future studies using CRISPR/Cas9-mediated complete NR4A1 knockout will help clarify the relative contribution of NR4A1-dependent and -independent mechanisms.

### 4.5. Limitations of the Study

Several limitations of this study should be acknowledged. First, while AAV8-mediated gene silencing achieved approximately 70–80% knockdown efficiency (NR4A1 expression reduced to 18–27% of control levels), this represents partial rather than complete loss of NR4A1 function. Complete knockout using CRISPR/Cas9 technology would provide more definitive evidence for the essential role of NR4A1 in mediating vitexin’s effects. Second, the absence of an empty AAV8 vector control group (AAV8-null or AAV8-scramble) precludes definitive exclusion of potential nonspecific effects arising from viral particle administration. Although AAV vectors are generally considered immunologically inert, and transcriptomic analyses have confirmed that rAAV8 does not activate monocyte-derived dendritic cells [53], the inclusion of empty vector controls in future studies is warranted. Third, the pharmacokinetic profile and oral bioavailability of vitexin were not assessed in this study. Vitexin is known to have low water solubility and limited oral bioavailability, which may affect its in vivo efficacy [50]. Fourth, this study was conducted exclusively in female ICR mice, and while this strain is widely used for PCOS modeling due to its high sensitivity to DHEA induction and robust reproductive performance, the translational relevance to human PCOS patients requires further validation. Finally, the relatively short intervention period (28 days) precludes assessment of long-term safety and efficacy.

### 4.6. Future Directions and Clinical Translation Prospects

Currently, vitexin has not been approved for clinical PCOS treatment. This study provides a mechanistic foundation for future translational research. Several steps are necessary before clinical trials can be considered. First, comprehensive pharmacokinetic and toxicological evaluations of vitexin in rodent and non-rodent models are required to establish safety profiles and optimal dosing regimens. Second, formulation strategies to improve oral bioavailability, such as nanoparticle-based delivery systems or phospholipid complexes, should be explored [50]. Third, the therapeutic efficacy of vitexin should be validated in additional animal models, including letrozole-induced PCOS models and high-fat diet-fed models that better recapitulate the metabolic features of human PCOS. Fourth, ex vivo studies using human ovarian tissues or primary granulosa cells from PCOS patients would help confirm the translational relevance of the NR4A1/NLRP3 pathway. Importantly, clinical studies have demonstrated that Vitex agnus-castus extracts, which contain vitexin as a major active component, show favorable tolerability and therapeutic effects in PCOS patients, including improved menstrual cyclicity, reduced hyperandrogenism, and increased pregnancy rates [19]. A recent randomized controlled trial confirmed the efficacy of Vitex agnus-castus in improving clinical and paraclinical parameters in PCOS patients. These clinical observations, combined with our mechanistic findings, support the potential of vitexin as a therapeutic agent for PCOS-associated ovarian fibrosis. Future studies should also validate NR4A1 as a therapeutic target in human ovarian tissues and assess long-term reproductive and metabolic outcomes following vitexin administration.

## 5. Conclusions

In conclusion, this study demonstrates that vitexin effectively ameliorates ovarian fibrosis in DHEA-induced PCOS mice. The protective effects of vitexin are associated with the restoration of NR4A1 expression and subsequent suppression of NLRP3 inflammasome activation, leading to reduced production of pro-inflammatory cytokines (IL-1β, IL-18) and downregulation of fibrotic markers (α-SMA, CTGF, Collagen1). NR4A1 gene silencing partially abolished these protective effects, confirming that the anti-fibrotic action of vitexin is at least partially dependent on NR4A1. Additionally, vitexin improves PCOS-associated metabolic abnormalities, including weight gain, hyperglycemia, dyslipidemia, and glucose intolerance. These findings provide the first experimental evidence linking vitexin to the NR4A1/NLRP3 signaling axis in the context of PCOS-associated ovarian fibrosis. While further pharmacokinetic, toxicological, and clinical studies are warranted, our results suggest that vitexin represents a promising therapeutic candidate for the treatment of PCOS and its long-term fibrotic complications.

## Figures and Tables

**Table 1:** Primer Sequences.

Primer Name	Forward Primer (5′-3′)	Reverse Primer (5′-3′)
ASC	GACAGTACCAGGCAGTTEGT	GAGTCCTTGCAGGTCAGGTT
IL-18	CACCTCAGACAACTGCCACT	GGCCAAATCACATCCAGTGC
α-SMA	TGAGCGTGGCTATTCCTTCG	AGCGTTCGTTTCCAATGGTG
NLRP3	CCCAGACCTCCAAGACCACTACG	CATCCGCAGCCAGTGAACAGAG
Smad3	TCGTCCATCCTGCCCTTCACC	ACTTCTCCTCCTGCCCGTTETG
CTGF	CACCGCACAGAACCACCACTC	AATGGCAGGCACAGGTCTTGATG
IL-1β	TCGCAGCAGCACATCAACAAGAG	AGGTCCACGGGAAAGACACAGG
NR4A1	GTCCGCTCTGGTCCTCATCAC	GGTCTCCTGCCACGGTAGC
Collagen1	ACATGTTCAGCTTTGTGGACCTC	GACAGTCCAGTTCTTCATGCACT
Iκbα	GCTGAAGCCGAGAGAGGAAC	TCCAGCACACAAGGCACTTC
TNF-α	CCCTCACACTCAGATCATCTTCTC	GCTACGACGTGGGCTACAG

## Data Availability

The original contributions presented in this study are included in the article. Further inquiries can be directed to the corresponding authors.
